# Molecular Evidence for Multiple Origins of the European Spined Loaches (Teleostei, Cobitidae)

**DOI:** 10.1371/journal.pone.0144628

**Published:** 2016-01-04

**Authors:** Anabel Perdices, Joerg Bohlen, Vendula Šlechtová, Ignacio Doadrio

**Affiliations:** 1 Department of Biodiversity and Evolutionary Biology, Museo Nacional Ciencias Naturales, CSIC, Madrid, Spain; 2 Laboratory of Fish Genetics Institute of Animal Physiology and Genetics, Libechov, Czech Republic; SOUTHWEST UNIVERSITY, CHINA

## Abstract

We present a phylogenetic investigation of the Northern Clade, the major monophyletic clade within the freshwater fish family Cobitidae, one of the most prominent families of freshwater fishes found in Asian and European waters. Phylogenetic reconstructions based on the cytochrome *b* and RAG-1 genes show the genera *Microcobitis*, *Sabanejewia*, *Koreocobitis* and *Kichulchoia* as monophyletic groups. These reconstructions also show a *Cobitis* sensu lato and a *Misgurnus* sensu lato group. The *Cobitis* sensu lato group includes all species of *Cobitis*, *Iksookimia*, *Niwaella* and *Kichulchoia*, while the *Misgurnus* sensu lato group includes *Misgurnus*, *Paramisgurnus* and *Koreocobitis*. Although the monophyly of both the *Cobitis* sensu lato and *Misgurnus* sensu lato groups is supported, relationships within the groups are incongruent with current generic definitions. The absence of monophyly of most genera included in the *Cobitis* sensu lato group (*Cobitis*, *Iksookimia* and *Niwaella*) or their low genetic differentiation (*Kichuchoia*) supports their consideration as synonyms of *Cobitis*. Molecular phylogenies indicate that the Asian species of *Misgurnus* experienced a mitochondrial introgression from a lineage of *Cobitis*. We also find two nuclear haplotypes in the same *Cobitis* species from the Adriatic area that, in the absence of morphological differentiation, may indicate molecular introgression. Most lineages within the Northern Clade consist of species found in East Asia. However, some lineages also contain species from Europe and Asia Minor. The phylogenetic relationships presented here are consistent with previous studies suggesting an East Asian origin of the Northern Clade. According to the current distributions and phylogenetic relationships of the *Misgurnus* sensu lato and *Cobitis* clade lineages, particularly of *M*. *fossilis* and *C*. *melanoleuca*, the range expansion of East Asian species into Europe was most likely via Siberia into Northern and Central Europe. Phylogenetic analyses also show that the *Cobitis* sensu lato group consists of two clear subgroups (I and II), each presenting geographical differences. Subgroup I is distributed exclusively in East Asian drainages with an Eastern European offshoot (*C*. *melanoleuca*), whereas Subgroup II includes species widespread throughout Europe (including the Mediterranean), Asia Minor, the Black Sea and the Caucasus, with some lineages related to species restricted to East Asia.

## Introduction

Determining how and when primary freshwater fishes (FWF) reached Europe and the Mediterranean peninsulas has been the focus of many biogeographic studies (e.g. [1], [2], [3], [4]). The fossil record and a high level of diversity observed in East Asian waters have been cited as evidence for an Asian origin of European FWF ([5], [6], [7]). Two major biogeographical hypotheses, the Northern dispersal (for a review, see [8]) and the Lago Mare [7], both postulate an initial expansion of FWF from East Asia across Siberia but differ on the origin of Mediterranean FWF. In the Northern dispersal hypothesis, Mediterranean FWF are ancestrally derived from Central European FWF, whereas in the Lago Mare hypothesis, Mediterranean FWF evolved from FWF inhabiting the Paratethys area (current Black Sea area). Thus, the Northern dispersal theory explains the current endemicity of Euro-Mediterranean FWF by their isolation from Central-European fishes due to the presence of old mountains. In the Lago Mare theory, the isolation of Euro-Mediterranean FWF is explained by rapid dispersal from the Paratethys area, favoured by decreased salinity levels during the “Lago Mare” phase of the Messinian salinity crisis—approximately 5 Million Years Ago (MYA).

The order Cypriniformes is a FWF group found throughout East Asia, Europe and peri-Mediterranean areas. Although Cypriniformes contains a number of polyploid groups that have undergone genome duplications, having clear implications for their phylogenies and systematics [9], the ubiquity and diversity of Cypriniformes have made this order one of the most studied FWF groups. In recent years, molecular phylogenies of some cypriniform families, for example, Cyprinidae, have been widely used in biogeographical studies [10], [11], [12], [13]. In a recent and comprehensive biogeographical study (mitochondrial and nuclear data of all groups of Leuciscinae), both major biogeographical hypotheses for peri-Mediterranean colonisation were partially supported [3].

Until recently, most biogeographical hypotheses proposed for FWF have been based on the Cyprinidae, with conclusions drawn from those studies extended to other primary FWF groups. Among cypriniforms, the family Cobitidae (spined loaches) is one of the most prominent families of FWF in Asian and European waters. However, cobitids are rarely investigated in broad biogeographical studies, likely due to the extensive taxonomical confusion related to cobitid species, and the occurrence of polyploid species and/or genera and frequent hybridisation events. Spined loaches are easily identifiable by their elongated body shape, a movable suborbital spine, and male sexual dimorphism, i. e. the presence of pectoral fin structures or body swelling. Early classifications of the spined loaches relied on these secondary sexual characters or body coloration to define taxonomic boundaries [14], [15]. These classifications were not phylogenetically based, and the presence or absence of characters was considered indicative of generic or specific differences, depending on the number of shared characters. However, in recent years, several phylogenetic studies have been published for the family Cobitidae [16], [17], [18] and more specifically, for some genera of spined loaches, i.e. *Cobitis* [19], [20], [21], *Sabanejewia* [22] and *Misgurnus* [23], [24], [25]. In these studies, some diagnostic characters of spined loaches were determined to be poor phylogenetic characters, for instance the absence of secondary sexual characters in *Kichulchoia* and *Niwaella* or pigmentation patterns in *Iksookimia*. Although the monophyly of the family Cobitidae was supported in several of these studies [26], resolution at the generic level was less supported [17], [27], [28].

A recent molecular phylogeny for the family Cobitidae divided the group into several lineages that are allopatrically distributed [17]. According to this study, the family Cobitidae consists of Southern lineages including eight nominal genera occurring in South and Southeast Asia and a Northern Clade that consists of species inhabiting the remaining parts of Asia and Europe, including all members of the genera *Cobitis*, *Kichulchoia*, *Koreocobitis*, *Iksookimia*, *Microcobitis*, *Misgurnus*, *Niwaella*, *Paramisgurnus* and *Sabanejewia*. The Southern lineages did not form a monophyletic group whereas the Northern Clade was monophyletic in all analyses. However, within the Northern Clade, most genera, namely *Cobitis*, *Iksookimia*, *Misgurnus* and *Niwaella*, were polyphyletic, and incongruities between nuclear and mitochondrial data were explained as mitochondrial introgression, at least for *Misgurnus* and *Cobitis* [17], [25], [28]. Lack of support for other taxa in the Northern Clade has lead to the recognition of a *Cobitis* sensu lato group that currently includes species of *Cobitis*, *Iksookimia*, *Niwaella* and *Kichulchoia* [17].

In this study, we focus on the Northern Clade with special emphasis on the *Cobitis* sensu lato group. We explore the potential impact of mitochondrial introgression on species and genera delimitation by comparing mitochondrial and nuclear gene genealogies using cytochrome *b* (cyt *b*) and recombination activating gene 1 (RAG-1). We use previously published sequences, primarily from East Asian species, together with new sequence data to encompass all known genetic diversity, to generate robust phylogenies that will allow identification of potential hybrid lineages. The broad distribution of some Northern Clade genera, particularly of the *Cobitis* sensu lato group across the Palearctic, raises questions about patterns of expansion across vast areas. The current discontiguous distribution of cobitids in East Asia and Europe is hypothesised to be the result of western preglacial dispersal by members of the eastern cobitid lineages via drainage connections in Central Europe prior to the Pleistocene glaciations (North dispersal hypothesis and Lago Mare dispersal theory). We use the combined gene phylogenies to test whether the presence of different *Cobitis* species in the Mediterranean peninsulas (Iberian, Italian and Western Balkan) is consistent with a scenario of a Central European colonisation (North dispersal hypothesis), or with a scenario of colonisation from the Paratethys area (Caucasus, Black Sea, Asia Minor), as suggested in the Lago Mare dispersal theory.

## Methods

### Ethics Statement

The investigation was conducted in accordance with ethical standards and Spanish legislation. Approval from the Ethics Committee was not necessary as wild fauna is excluded in LAW 32/200 of 7th November 2007 (BOE 8/11/2007), which regulates the use of animals in experiments in Spain. No endangered species were used. Sampling was conducted by electrofishing, and the specimens were sacrificed by over-anesthesia with MS-222 (tricaine methanesulfonate) and/or preserved in 95% ethanol in the field. Permission for sampling in Spanish waters was issued by the Ministry of Agriculture, Forestry and Water Resources of Spain. Permission for sampling in Greek and Romanian waters was issued by the Ministries of Agriculture of Greece and Romania, respectively.

### Taxon sampling

Spined loaches of the Northern Clade were widely collected from the distribution area from East Asia (including Vietnam) to Europe and North Africa (Morocco), including the peri-Mediterranean area, Asia Minor and the Black Sea area and adjacent countries. We sequenced mitochondrial cytochrome *b* (cyt *b*) (1140 base pairs [bp]) and nuclear recombination activating gene RAG-1 (exon 3, 897 bp). The taxa used in [19], [21], [22] and herein, were also sequenced for RAG-1. We analysed the new sequences together with previously published sequences for phylogenetic analyses. New sequences (a total of 141) were deposited in GenBank (KP11080-KP161204, KT717937-KT717952) (S1 Appendix).

We analysed 87 out of the 99 species (90% coverage) of the nine genera included in the Northern Clade [17], [29]. When possible, we analysed the same specimens for both genes. We used *Pangio* and *Kottelatlimia* from the Southern lineages as outgroups [17]. We constructed three independent datasets. The first two datasets consisted of the mitochondrial (N = 204; cyt *b* dataset) and nuclear (N = 168; RAG-1 dataset) genes to check for molecular incongruences and possible hybridisation events. In the cyt *b* dataset, we included many of the East Asian *Cobitis* species that were used for phylogenetic assessment of East Asian taxa. In the third dataset (combined dataset), we combined mitochondrial and nuclear sequences (N = 140) (taxa with conflicting phylogenetic positions were removed including *Misgurnus anguillicaudatus*, *Misgurnus mohoity* and some *Cobitis* sp. B specimens; see results and discussion) to construct a robust phylogenetic hypothesis to delimit taxa and test previously proposed biogeographical hypotheses. All taxa have the same number of codons for cyt *b* and RAG-1 with no stop codons in the translated amino acid sequences. Observed divergences were based on uncorrected p-distances.

### Molecular analysis

Total DNA was extracted from ethanol-preserved fin or white muscle tissues either using standard phenol/chloroform methods [30], including a proteinase K digestion step, or the Charge Switch gDNA Microtissue kit (Invitrogen, Inc.), according to the manufacturer’s protocol. The entire cyt *b* gene (1140 bp) was PCR amplified using two sets of primers: GluDGL [31] and H16460 and Glu-L.Ca14337-14359 and Thr.-H.Ca15568-15548 [16]. A fragment of the nuclear RAG-1 gene (897 bp) was PCR amplified using primers RAG1-1F [32] and RAGRV1 [17]. Both genes were amplified in a total volume of 25 μl using conditions described in [33].

Chromatograms and alignments were verified with Sequencher ver. 4.0 (Gene Codes Corporation, Inc.). Nucleotide composition was examined for variable sites, and a χ^2^ homogeneity test of base frequencies for all positions was checked using PAUP* 4.0a123 [34]. Nucleotide saturation was analysed by plotting uncorrected p-distances at first, second and third codon positions against absolute distance values. Relationships between genotypes were resolved by distance methods with Sequencer 6.1 (written by B. Kessing). For phylogenetic reconstructions, all three datasets were analysed by Bayesian inference (BI) using MrBayes 3.1.2 [35], [36] and a maximum likelihood (ML) method as implemented in RAxML-HPC [37] and its graphical interface raxmlGUI 1.3 [38]. We determined the best-fit model of molecular evolution for each gene dataset and the combined dataset using the Akaike Criterion (AIC) in jModeltest ver 2.1.4 [39]. MrBayes was run with 6 substitution types (nst = 6) and considered gamma-distributed rate variation and the proportion of invariable positions (GTR + G + I) for the cyt *b*, RAG-1 and combined datasets (independently analysed by gene). For the cyt *b* and combined datasets, a partition by codon position was also taken into account for the cyt *b* gene. For BI, we ran four simultaneous Monte Carlo Markov Chains for 4 million generations, sample frequency every 1000 generations, chain temperature 0.2. Log-likelihood stability was attained after 10,000 generations, and we excluded the first 1,000 trees as burn-in. The remaining trees were used to compute a 50% majority rule consensus tree in PAUP*. For ML analyses, we conducted heuristic searches (1000 runs) under a GTR + I + G model for the cyt *b* and RAG-1 datasets and for the different codon positions for the cyt *b* gene. For the combined dataset, ML analyses were conducted under a GTR + I + G model for the partitioned dataset (by gene and by codon positions for cyt *b*). Robustness of inferred trees was assessed by bootstrapping (1000 replicates) in ML analyses [40] and posterior probability values in BI analyses.

## Results

Sequence data for the mitochondrial cytochrome *b* (1140 bp) and nuclear RAG-1 (897 bp) genes were analysed independently for 204 and 168 individuals, respectively. Nucleotide frequencies were not significantly different across taxa (cyt *b* χ^2^ = 271.96, p = 1.0 df = 591; RAG-1 χ^2^ = 19.24, p = 1.0, df = 501). Nucleotide composition analysis of the two genes showed similar proportions per nucleotide for RAG-1 (23.6–27.69%) but an anti-G bias (15.2%) for cyt *b*. The cyt *b* gene contained more phylogenetically informative positions (43.5%) compared to RAG-1 (22.4%). Saturation was not observed for cyt *b* or RAG-1 sequences (plots not shown).

**Fig 1:**
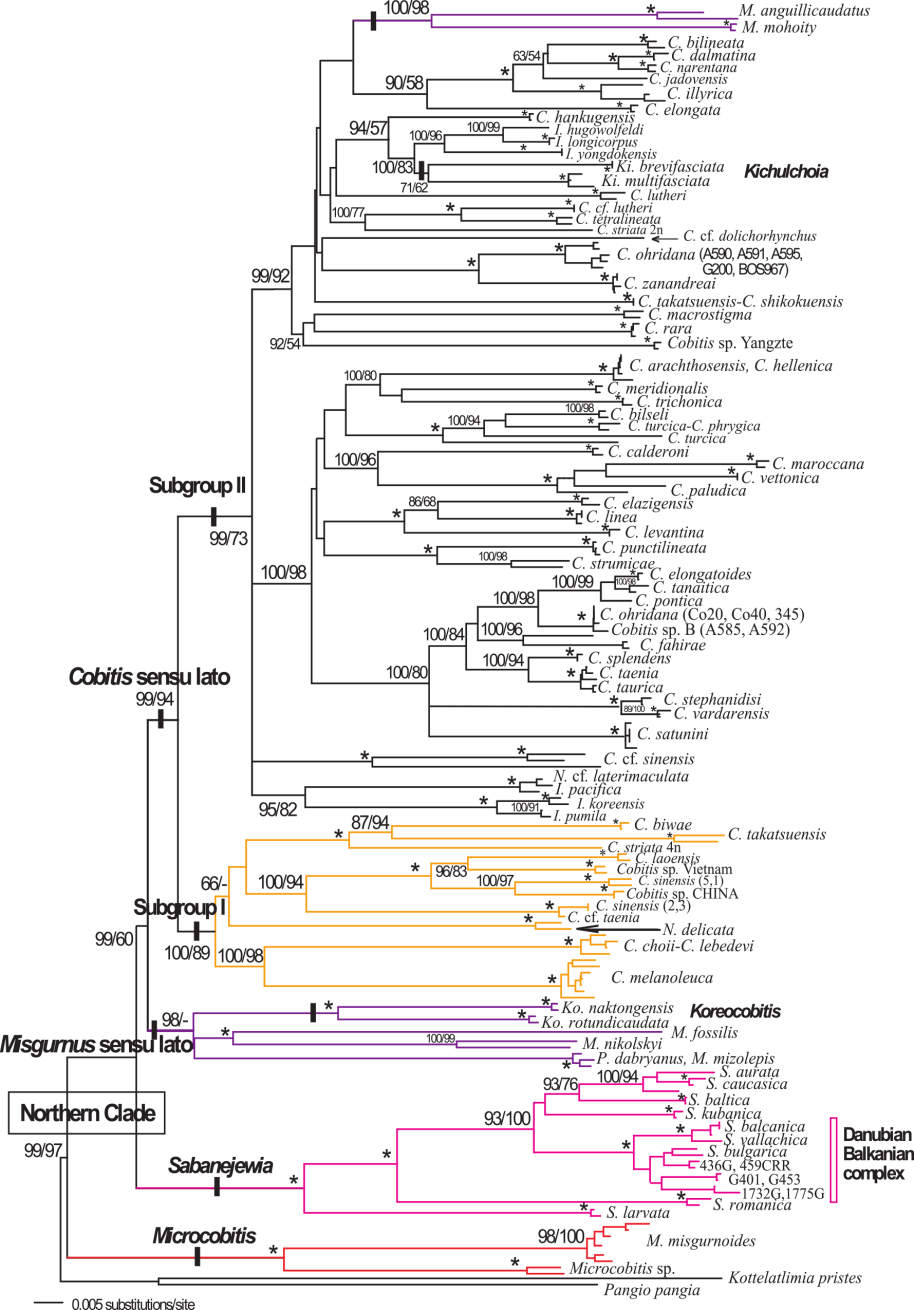
Phylogenetic relationships of the Northern Clade based on the cyt *b* dataset (N = 204). Numbers on branches represent the posterior probabilities for BI (x100) and bootstrap values for ML (1000 replicates), respectively. Asterisks indicate values that are 100%; (-) indicate the branch was not supported. Major Northern Clade lineages based on the mitochondrial sequences are highlighted with different branch colours, which are maintained in Figs 2–4.

**Fig 2:**
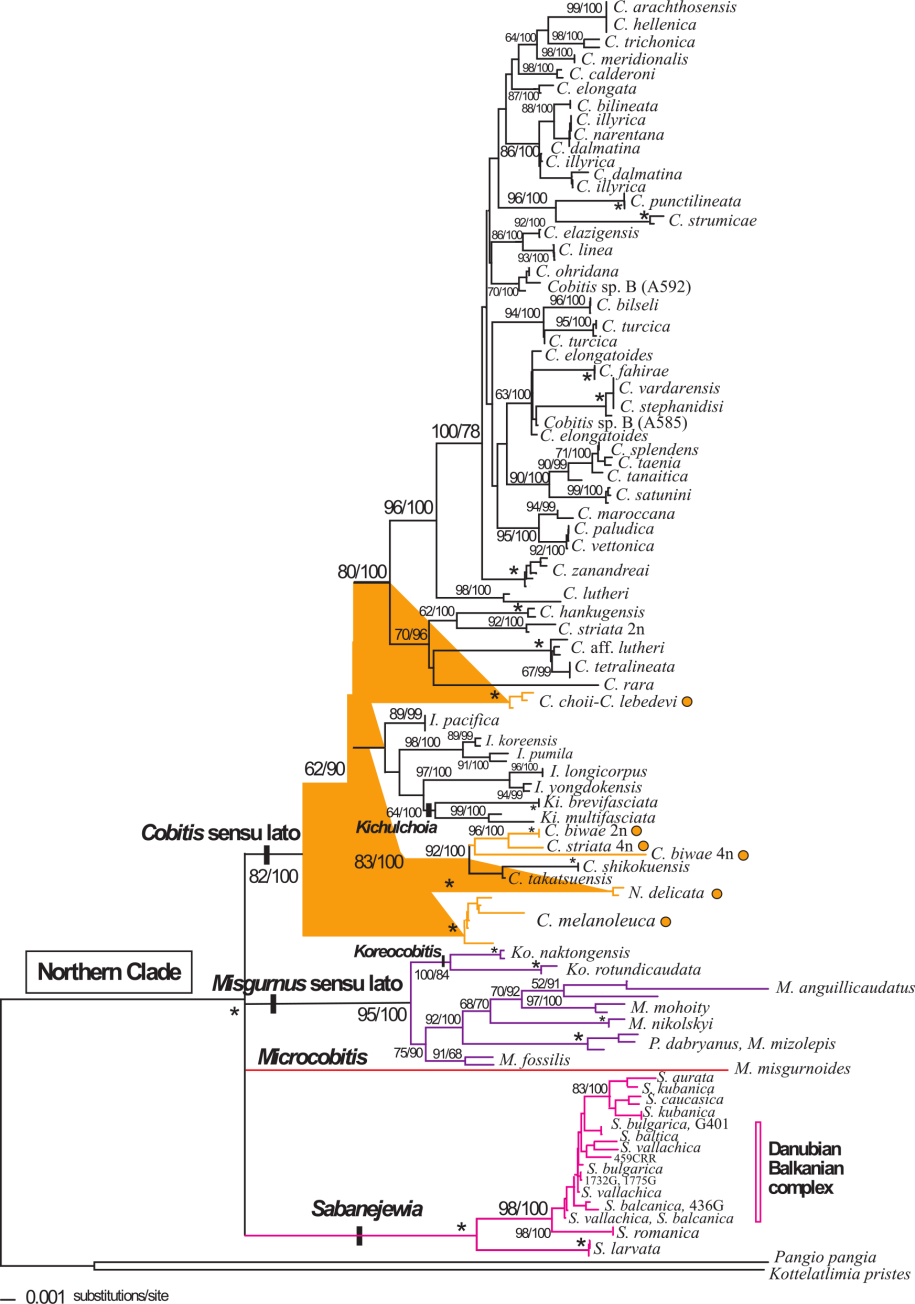
Phylogenetic relationships of the Northern Clade based on the RAG-1 dataset (N = 168). Numbers on branches represent the posterior probabilities for BI (x100) and bootstrap values for ML (1000 replicates), respectively. Asterisks indicate values that are 100%; (-) indicate the branch was not supported. Branch colours represent major Northern Clade lineages obtained with the mitochondrial (cyt *b*) dataset as shown in Fig 1. Orange dots indicate taxa belonging to Subgroup I of the *Cobitis* sensu lato group recovered with the cyt *b* dataset.

Independent analyses for the mitochondrial and nuclear genes were overall in agreement (Figs 1 and 2). Not all species were available for the nuclear analysis, and in general, the nuclear phylogeny was less resolved than the mitochondrial phylogeny. The Northern Clade was a well-supported monophyletic group (>97%), as were the genera *Sabanejewia* (100%) and *Microcobitis* (100%). However, several incongruities were observed between mitochondrial and nuclear phylogenies (Figs 1 and 2). For example, in the mitochondrial analyses, the genus *Misgurnus* was never monophyletic. These analyses supported the separation of *Misgurnus* species into two independent lineages: *M*. *anguillicaudatus* and *M*. *mohoity* clustering with the *Cobitis* species, and *M*. *fossilis*, *M*. *mizolepis* and *M*. *nikolskyi* clustering with *Paramisgurnus dabryanus* and *Koreocobitis naktongensis* and *K*. *rotundicaudata* (Fig 1). In contrast, a strongly supported clade (>95%) that included all *Misgurnus* (including *M*. *anguillicaudatus* and *M*. *mohoity*) and *Koreocobitis* species was observed in the nuclear analyses. We refer to this group as the *Misgurnus* sensu lato group. Also in the nuclear phylogeny, *Cobitis*, *Iksookimia*, *Kichulchoia* and *Niwaella* species were included in a larger clade, the *Cobitis* sensu lato group, with moderate to high support in all analyses (82–100%) (Figs 1 and 2). We found another incongruity between phylogenies, specifically in the *Cobitis* sensu lato group. Specimens of Adriatic *Cobitis* sp. B had a single mitochondrial haplotype but two nuclear haplotypes: one specimen (A592) appears more closely related to *C*. *ohridana* and the other (A585) to European and Mediterranean species, such as *C*. *elongatoides*. Specimens of *Cobitis* sp. B (A585 and A592) and the species *M*. *anguillicaudatus* and *M*. *mohoity* were excluded from the combined analyses.

The mitochondrial phylogeny included many East Asian *Cobitis* species that were not available for nuclear analyses, namely *Cobitis* sp. CHI (China), *C*. cf. *taenia*, *C*. cf. *granoei*, *C*. *laoensis*, *C*. *sinensis* and *Cobitis* sp. Vietnam. These species, as part of the *Cobitis* sensu lato group, formed a monophyletic group with most of the other East Asian *Cobitis* species analysed (e.g. *C*. *biwae*, *C*. *choii*, *C*. *lebedevi*, *C*. *melanoleuca*, *C*. *striata* and *C*. *takatsuensis*) as well as with *N*. *delicata* from Japan.

**Fig 3:**
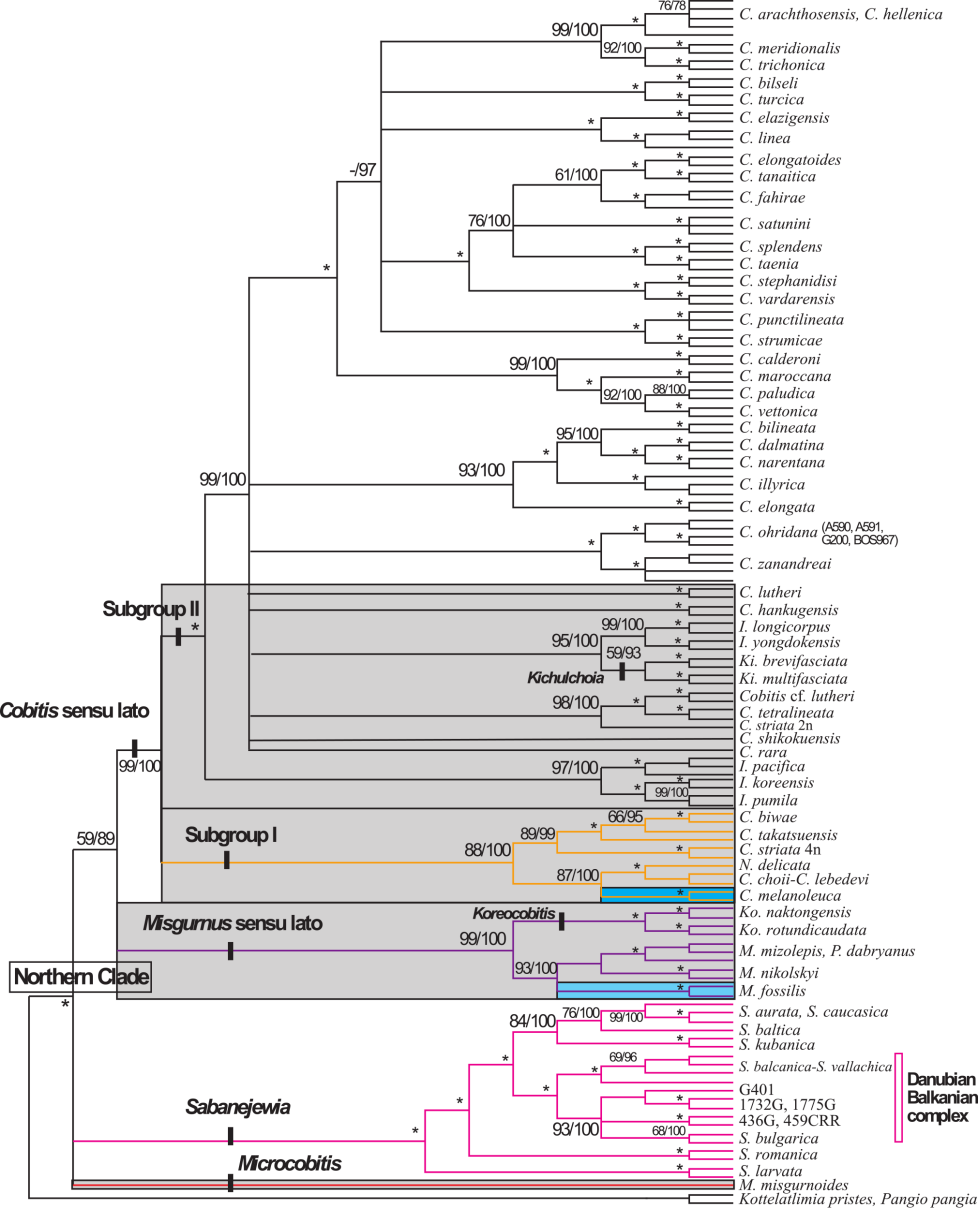
Phylogenetic relationships of the Northern Clade based on the combined dataset of cyt *b* and RAG-1 sequences (N = 140). Values on branches correspond to values for BI (x100) and bootstrap values for ML (1000 replicates). Asterisks indicate values that are 100%; (-) indicate the branch was not supported. East Asian lineages indicated in grey boxes with species with European distributions highlighted in blue boxes. Branch colours represent major Northern Clade lineages obtained with the mitochondrial (cyt *b*) dataset as shown in Fig 1.

In all analyses of the combined dataset (N = 140), the Northern Clade was monophyletic (100%) as were the genus *Sabanejewia* and the *Misgurnus* and *Cobitis* sensu lato groups (>99%). Although only one *Microcobitis* specimen was used in the combined analyses, the mitochondrial phylogeny shows that all *Microcobitis* specimens form a monophyletic lineage consisting of two well-differentiated genetic groups (mean p distance = 19.1%, range 15.8–22.4%). In the combined analyses, species of the genus *Sabanejewia* formed a monophyletic group with most having well supported, resolved relationships (Fig 3). *Sabanejewia larvata* and *S*. *romanica* were the most differentiated species (Fig 3), whereas *Sabanejewia* species from the Danubian-Balkanian complex were the sister group of the Caucasian and Baltic group. Species of the genera *Misgurnus*, *Paramisgurnus* and *Koreocobitis* formed a monophyletic group with high support (>99%) (Fig 3). Within the *Cobitis* sensu lato group, none of the described genera formed a monophyletic group. *Cobitis*, the most species-rich genus of the family Cobitidae, was polyphyletic in all analyses. Our analyses further suggest a close relationship between the genera *Cobitis*, *Kichulchoia*, *Iksookimia* and *Niwaella*, with the latter three being nested within the genus *Cobitis* (Figs 1–3). The *Cobitis* sensu lato group was subdivided into two subgroups (Subgroups I and II). Subgroup I contained East Asian *Cobitis* species from Japan and the Russian Far East including *C*. *biwae*, *C*. *choii*, *C*. *lebedevi*, *C*. *striata* (4n), *C*. *takatsuensis*, the East Asian—European species *C*. *melanoleuca* and *N*. *delicata* from Japan (type species of the genus *Niwaella*). Subgroup II included several differentiated lineages consisting of the majority of species assigned to *Cobitis*, *Iksookimia*, *Kichulchoia* and *Niwaella* from Europe, Asia Minor, the Black Sea, the Caucasus, Korea (except *C*. *choii*) and Japan (*C*. *striata* (2n), *C*. *shikokuensis*) (Figs 1 and 3). Many of these lineages contained only one species, e.g. *C*. *lutheri*, *C*. *rara*, *C*. *shikokuensis*, or a few species, such as i) *I*. *koreensis*, *I*. *pumila* and *I*. *pacifica*, ii) *K*. *brevifasciata*, *K*. *multifasciata*, *I*. *longicorpus*, *I*. *yongdokensis*, iii) *C*. *tetralineata*, *C*. *striata* 2n, *C*. cf. *lutheri* and iv) *C*. *ohridana* and *C*. *zanandreai*. In another lineage, other *Cobitis* species from the Adriatic area (*C*. *bilineata*, *C*. *dalmatina*, *C*. *illyrica* and *C*. *narentana*) were related to *C*. *elongata* (Danubian). The remaining *Cobitis* species from Europe (including the Iberian and Balkan peninsulas), Asia Minor and the Black Sea area grouped together in a lineage. It is noteworthy that *Cobitis* species inhabiting Japan, Korea and the Adriatic area did not cluster together, but rather were divided between the two subgroups (Figs 1–3). Pairwise divergences within Subgroup II (0–1.73% with a mean of 0.28±0.38%) were much lower than those within Subgroup I (0–9.66% with a mean of 1.6±3.56%).

## Discussion

### Phylogeny and systematic implications

Our findings confirm the monophyly of the *Cobitis* sensu lato group (*Cobitis*, *Iksookimia*, *Kichulchoia* and *Niwaella*), as previously suggested [17]. According to our results, there is no correlation between morphological and molecular-based phylogenies in the *Cobitis* sensu lato group. Species with similar morphological characters traditionally used in cobitid systematics, i.e. secondary sexual characters and pigmentation patterns, do not form monophyletic lineages. For example, the genus *Iksookimia* as identified by diagnostic pigmentation patterns appears polyphyletic in all analyses. Moreover, some *Iksookimia* species appear, at the mitochondrial level, more closely related to other groups endemic to the southern Korean peninsula, e.g. *Kichulchoia*, and *C*. *hankugensis*. However, the relationship between *Iksookimia* species and *C*. *hankugensis* is not supported in nuclear phylogenies. Different evolutionary speeds of markers or hybridization, as previously found for *I*. *longicorpa* and *C*. *takatsuensis* [41], may account for differences between nuclear and mitochondrial markers.

Other genera characterised by the absence of secondary sexual dimorphism are *Niwaella* and *Kichulchoia*. Despite analysing only a few species (and specimens) of *Niwaella*, our molecular data do not support this genus as a natural group. In contrast, the genus *Kichulchoia* was always monophyletic with *K*. *brevifasciata* and *K*. *multifasciata* as sister species. However, the phylogenetic data suggest that *Niwaella* and *Kichulchoia* may be morphologically specialised species derived from a local *Cobitis* species rather than evolutionarily distinct genera. For *Kichulchoia*, *Niwaella* and *Iksookimia* to be considered distinct genera (i.e. monophyletic group), extensive generic rearrangements would be required for *Cobitis* species lacking specific morphological diagnostic characters. We therefore propose *Kichulchoia*, *Niwaella* and *Iksookimia* as synonyms of *Cobitis* and consider all species of the *Cobitis* sensu lato lineage as belonging to the genus *Cobitis*. Further morphological, karyological and molecular studies of the genus *Cobitis* would greatly aid the identification of groups included in this genus.

In this study, using the cyt *b* dataset, we analysed many of the currently recognised *Cobitis* species (50 out of 66, [29]). Our findings show most *Cobitis* species as monophyletic lineages. However, in some cases, morphologically similar species from geographically contiguous areas, which we consider young species, have not reached reciprocal monophyly, e.g. *C*. *arachthosensis-C*. *hellenica*. Our mitochondrial data confirm the presence of many undescribed forms of *Cobitis* in East Asia, particularly in China and Taiwan. It also confirms the inclusion of multiple lineages under the species name *C*. *sinensis* (Fig 1) that might actually represent different species, as previously suggested [28], [42].

### Molecular introgression

Molecular introgression at the mitochondrial level may explain phylogenetic incongruences found in the genera *Cobitis* and *Misgurnus* [17], [18], [25]. In the mitochondrial (cyt *b*) phylogeny, *M*. *anguillicaudatus* and *M*. *mohoity* together form a monophyletic lineage within the *Cobitis* sensu lato group whereas *M*. *nikolskyi*, *M*. *fossilis*, *M*. *mizolepis* and *P*. *dabryanus* are more closely related to the genus *Koreocobitis*. However, in the nuclear (RAG-1) phylogeny, all *Misgurnus* species are included in a monophyletic clade with *Koreocobitis*, which we refer to as the *Misgurnus* sensu lato group. Following [17], we suggest that a mitochondrial introgression occurred from an ancestral species of *Cobitis* into an ancestral species of *Misgurnus*. The analysed samples of *P*. *dabryanus* and *M*. *mizolepis* are genetically very similar, a result supporting the hypothesis that *P*. *dabryanus* and *M*. *mizolepis* are conspecific [25], [29], [43], [44]. The diagnostic characters that separate *Paramisgurnus* from *Misgurnus* (e.g. an elongated vs. round *lamina circularis* and slightly larger body scales) are rather minor, and they share an apomorphy (a suborbital spine overgrown by skin). However, the relationships between the genera *Misgurnus* and *Paramisgurnus* differed in the two datasets (cyt *b* and RAG-1). Lacking a detailed comparison, we consider *M*. *mizolepis* genetically very close to *P*. *dabryanus*.

Our data suggest a genetic introgression of *Cobitis* species inhabiting the southeast Danube basin and *Cobitis* species inhabiting the Ohrid-Skadar lake system in the Mediterranean area, as previously noted [33], [45]. Although the cyt *b* and RAG-1 datasets do not robustly support a hybridisation event for *C*. *ohridana* with Central European *Cobitis*, we do find two mitochondrial types of *C*. *ohridana*. The first mitochondrial type is represented by specimens of *C*. *ohridana* that form a sister group with *C*. *zanandreai*. The second type is represented by *C*. *ohridana* specimens (including specimen Co20 from Lake Ohrid, type locality of the species) that are most closely related to *Cobitis* sp. B, a newly identified species [33], and that cluster with the Danubian and Eastern European species *C*. *elongatoides* and *C*. *tanaitica*. However, at the nuclear level, *Cobitis* sp. B contains two nuclear alleles: one represented by specimen A592, which is related to *C*. *ohridana*, and another represented by specimen A585, which is related to the Danubian species *C*. *elongatoides* and the Greek-Turkish species *C*. *stephanidisi*, *C*. *vardarensis* and *C*. *fahireae*. Both specimens showed some heterozygous positions with unclear assignment to any potential parental species. No morphological differences between the two *Cobitis* sp. B specimens have been observed (Bohlen unpubl. data). In the absence of karyological data, the presence of two nuclear haplotypes in the same species may be a case of introgression. However, for genetic introgression in *Cobitis* to be supported, further analyses based on more genes and ploidy data are needed to confirm this hypothesis. This molecular introgression is noteworthy because, according to a traditional biogeographical hypothesis, the Balkan Ohrid-Skadar lake system with its high level of FWF endemicity is an example of ancient European lakes [46], [47]. However, recent molecular studies contradict this hypothesis [48].

### Testing biogeographical hypotheses

**Fig 4:**
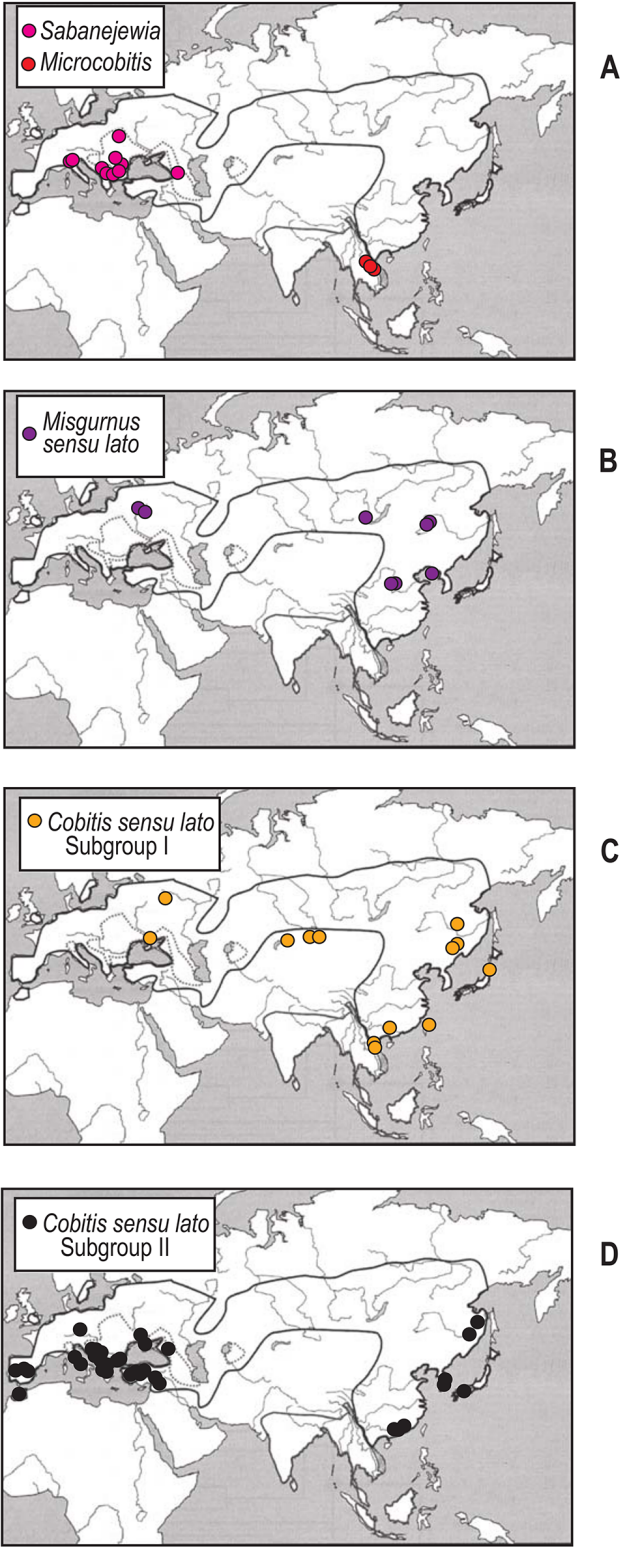
Distribution maps of the major lineages of the Northern Clade based on analysed specimens. General distribution of the Northern Clade based on Fig 8 in Šlechtová et al. (2008). A. Distribution map of analysed *Sabanejewia* and *Microcobitis* species. B. Distribution map of analysed *Misgurnus* sensu lato group species. C-D. Distribution maps of Subgroup I and II species of the *Cobitis* sensu lato group, respectively. Coloured dots on range reconstructions are the same as the branch colours used in Figs 1–3 to indicate different Northern Clade lineages.

Our discussion of Northern Clade biogeography focuses on the molecular lineages obtained from the cyt *b* and combined datasets (with conflicting lineages [*M*. *anguillicaudatus*, *M*. *mohoity* and *Cobitis* sp. B specimens A585 and A592] removed; see previous section) and current species ranges. Overall, the distributions of the four major Northern Clade lineages in East Asia, Europe and Asia Minor suggest an early radiation of this group in East Asia (Fig 4A–4D). The phylogenetic pattern observed suggests that *Microcobitis* is a very old lineage of the Northern Clade. Currently, *Microcobitis* is only known from one described species from Vietnam and one undescribed species from Laos [49]. Also, this group represents the only example of sympatric occurrence between the Northern Clade and southern lineages: the species ranges of the undescribed *Microcobitis* species from Laos and species of the genus *Lepidocephalichthys* from the southern lineages overlap. Our reconstruction indicates a temporal gap until the next speciation event that separated the *Sabanejewia* lineage. The genus *Sabanejewia*, with no current species in East Asia, represents the exception to the geographical pattern observed in the Northern Clade (Fig 4A). Basal *Sabanejewia* lineages are found in North Italy (*S*. *larvata*) and in a restricted area of the Danube (*S*. *romanica*); however, the fossil record (†*Sabanejewia shargaensis*) from West Mongolia and East Kazakhstan indicates a broader range of this genus in Western Asia during the Middle to Late Miocene [50]. Our phylogeny supports the monophyly of the Baltic, Caucasian and Caspian species, as the sister group of the Danubian-Balkanian lineage, which evolved during the Pliocene-Pleistocene [22]. The sister group of both these major lineages was the Danubian *S*. *romanica*, suggesting a Danubian ancestor. This finding is incongruent with those from most cyprinids, which are ancestrally related to Mesopotamian freshwater fishes or freshwater fishes inhabiting areas near the Black Sea and Caucasus Mountains [1], [3], [51].

We observe a major East Asia-Europe disjunction within the *Misgurnus* sensu lato clade (Fig 4B). The distribution areas of the East Asian species of *Misgurnus* and *Koreocobitis* versus that of the European *M*. *fossilis* (pp 99%) illustrate this disjunction. The current distribution of *M*. *fossilis* exclusively in Northern European waters and its absence in the Mediterranean peninsulas and the Caucasus and Asia Minor regions support the hypothesis that *M*. *fossilis* was a Northern Siberian migrant and did not colonise Europe via the Tethys Sea during the Messinian salinity crisis.

Another East Asian-European disjunction is found within the Cobitis sensu lato group (Fig 4C and 4D). This genus has a wide distribution area stretching from East Asia through Siberia to Europe and Northern Morocco (Africa) and includes the Caucasus, the Black Sea drainage, Asia Minor and the Mediterranean peninsulas. Our phylogenies show a strongly supported bifurcation of *Cobitis* into two major subgroups (I and II in Fig 1) that, for the most part, match the geographical distribution of species. However, there are some regions, such as China, Korea, Japan and the Russian Far East, that have species in both subgroups. Although both subgroups contain species distributed in East Asia and Europe, they exhibit important differences (Figs 3 and 4). Subgroup I is an East—Southeast Asian clade with an offshoot in Europe (*C*. *melanoleuca*), similar to *M*. *fossilis* but with a broader, more continuous distribution that encompasses the Russian Far East, Siberia and Northeast Europe (western limit is the Don River). *Cobitis melanoleuca* is absent from Central Europe, the Mediterranean peninsulas, the Caucasus and Asia Minor. Although *C*. *melanoleuca* split early from its East Asian relatives, its distribution and low level of molecular differentiation indicate that this species is a recent Northern Siberian immigrant in Eastern Europe, as previously suggested [52], [53]. A similar scenario has been described for two North Asian freshwater fishes (*Carassius gibelio* and *Rhynchocypris percnurus*), which also have East Asian relatives and populations with very low genetic differentiation across their range [52]. These evidences support the hypothesis of a postglacial European colonisation of these species from East Asia across Siberia into Northeast Europe. A recent molecular study of *C*. *melanoleuca* populations across its entire range indicates that the Siberian populations (which inhabit the central parts of the range) as the mitochondrial source for populations inhabiting the eastern and western extremes of the species range [53]. Overall, this evidence supports the divergence of the *Cobitis* sensu lato group in East Asia (Subgroup I) with an Eastern European offshoot (*C*. *melanoleuca*) that recently inhabited European waters via a northern route of expansion. Subgroup II consists of species distributed in East Asia, the Caucasus, the Black Sea, Asia Minor, Central Europe, the Mediterranean peninsulas and Morocco. All of the species from Europe (including the Mediterranean area), Morocco, the Black Sea, Asia Minor and the Caucasus included in this study are within this subgroup. However, some of these species also have relationships with lineages restricted to East Asia. The majority of the Mediterranean *Cobitis* species have restricted distributions replacing one another in the main Mediterranean drainages, suggesting a vicariant pattern of speciation. The phylogenetic and distribution patterns of the Mediterranean *Cobitis* species suggest there have been no adjacent range expansions in the Mediterranean area in the past, even when geological conditions were favourable, such as during the Messinian salinity crisis. However, for some East Asian members of Subgroup II, i. e. those from China, Korea, the Russian Far East and Japan, we found more than one monophyletic lineage for the same described species, namely *C*. *sinensis*, suggesting multiple origins of their ichthyofauna and/or multiple exchanges after secondary contact. In contrast to the widespread Asian distribution of the East Asian Subgroup I (of the *Cobitis* sensu lato group) with a single European offshoot, Subgroup II consists of several East Asian lineages but also includes species found widespread throughout Europe (including the Mediterranean region), which are closely related to Asia Minor, Black Sea and Caucasus species.

## Supporting Information

S1 Appendix Species identifications after [29]. Listed are the IDs for individuals as referred to in the corresponding listed reference, localities and accession numbers for the cyt *b* and RAG-1 sequences used in phylogenetic analyses. Country code abbreviations according to ISO alpha-2.
(DOC)
