# Correction: Molecular Evidence for Multiple Origins of the European Spined Loaches (Teleostei, Cobitidae)

**DOI:** 10.1371/journal.pone.0151228

**Published:** 2016-03-18

**Authors:** The PLOS ONE Staff

The image for Fig 2 is incorrect. The publisher apologizes for the error. Please see the corrected Fig 2 here.

**Fig 2:**
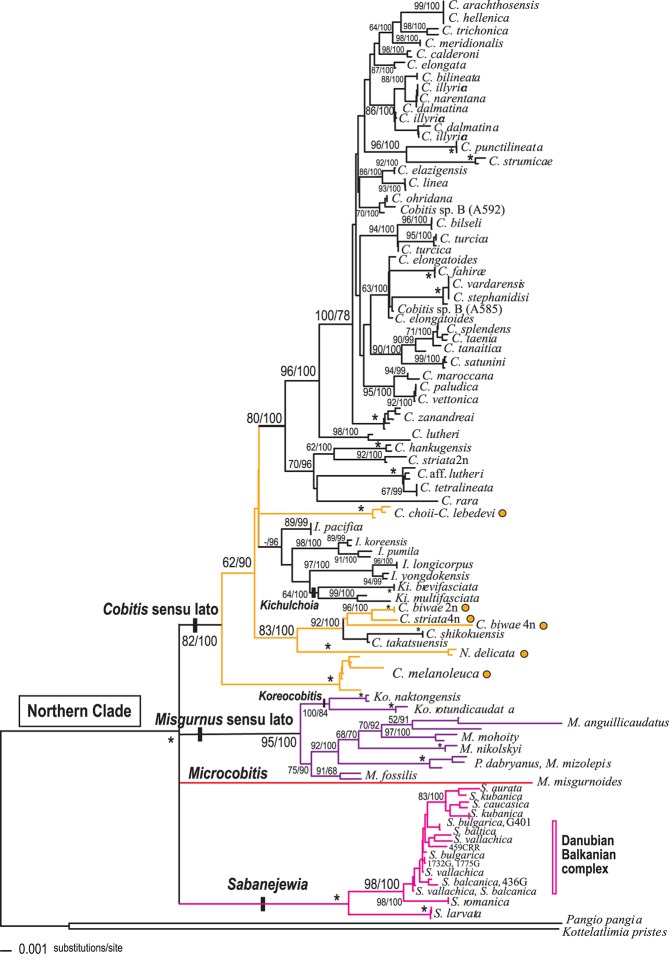
Phylogenetic relationships of the Northern Clade based on the RAG-1 dataset (N = 168). Numbers on branches represent the posterior probabilities for BI (x100) and bootstrap values for ML (1000 replicates), respectively. Asterisks indicate values that are 100%; (-) indicate the branch was not supported. Branch colours represent major Northern Clade lineages obtained with the mitochondrial (cyt *b*) dataset as shown in Fig 1. Orange dots indicate taxa belonging to Subgroup I of the *Cobitis* sensu lato group recovered with the cyt *b* dataset.
