# Supplementary material for: Molecular Evidence for Multiple Origins of the European Spined Loaches (Teleostei, Cobitidae)
Source: PLoS One. 2016 Jan 4;11(1):e0144628. doi: 10.1371/journal.pone.0144628 (PMC4699775; doi:10.1371/journal.pone.0144628)
Supplement: S1 Appendix — Listed are the IDs for individuals as referred to in the corresponding listed reference, localities and accession numbers for the cyt b and RAG-1 sequences used in phylogenetic analyses. Country code abbreviations according to ISO alpha-2. (DOC) [file pone.0144628.s001.doc]

| **Species (according to Kottelat 2012)** | **Species (not according to Kottelat, 2012)** | **ID** | **River, drainage, province, country** | **CYTB**  **Acc. Nº** | **RAG-1**  **Acc. Nº** | **Latitude** | **Longitude** | **Reference** |
| --- | --- | --- | --- | --- | --- | --- | --- | --- |
| *Cobitis arachthosensis* |  | 161G  1990G  1992G | R. Arachtos, Arachtos, Arta, GR  Arachtos channel, Arachtos, Keramates, GR  Arachtos channel, Arachtos, Keramates, GR | KP161080  KP161081  KP161081 | KP161136  KP161137  KP161138 | 39° 7'57.90"N 39° 8'6.92"N | 20°59'18.07"E 20°59'13.43"E |  |
| *Cobitis bilineata* |  | 79TA  274TA | R. Reno, Reno, Vergato, IT  R. Esino, Esino, Jesi, IT | EF672365  EF672361 | EF672416  KP161139 | 44°17'0.82"N  43°31'19.12"N | 11° 6'58.33"E  13°16'41.41"E | [33]  [33] |
| *Cobitis bilseli* |  | 21  215TK  362TR | Lake Beysehir, Beysehir, Konya, TR  Lake Beysehir, Beysehir, Konya, TR  Lake Beysehir, Beysehir, Konya, TR | DQ217391  DQ217392 | KP161140  KP161141  KP161142 |  |  | [19]  [19] |
| *Cobitis biwae* |  | A2237  190  Chr286 (4n)  Chr288 (2n)  Chr289 (2n) | R. Iwaki basin, Iwaki, Aomori, Honshu, JP  R. Iwaki basin, Iwaki, Aomori, Honshu, JP  R. Sonobe, Ooi R. basin, Kyoto, Honshu, JP  R. Kishida, Hyogo, Honshu, JP  R. Kishida, Hyogo, Honshu, JP | EF508495  KP161083 | AB531331  AB531336  AB531337 |  |  | [17]  [54]  [54]  [54] |
| *Cobitis calderoni* |  | 116ES  1252ES | R. Cega, Duero, Viana de Cega, ES  R. Arlanza, Duero, Castrillo de la Reina, ES | KP161084  KP161085 | KP161143  KP161144 |  |  |  |
| *Cobitis choii* |  | A2029 | R. Mi Ho, Geum, Yeo Cheong, KR | EF508510 | EF508622 |  |  | [17] |
| *Cobitis dalmatina* |  | BOS728B  BOS728C | R. Cetina, Cetina, Kosore, HR  R. Cetina, Cetina, Kosore, HR | EF672376  EF672377 | EF672419  KP161145 |  |  | [33]  [33] |
| *Cobitis cf. dolichorhynchus* |  | IHCAS18 | Unknown river, Hainan, CN | DQ105232 |  |  |  | [27] |
| *Cobitis elazigensis* |  | 372  A2094 | R. Muhrad Nehri, Euphates, Erzican, TR  R. Muhrad Nehri, Euphates, Erzican, TR | DQ217385  EF508496 | KP161146  EF056337 |  |  | [19]  [17] |
| *Cobitis elongata* |  | A2093  303CRE | R. Vit, Danube, Pleven, BG  R. Nera Danube, Sasca, RO | EF50849  AF263069 | EF056332  EF672420 |  |  | [17]  [21] [33] |
| *Cobitis elongatoides* |  | 95SAB  98SAB | R. TImis, Danube, Albina, RO  R. TImis, Danube, Albina, RO | AF263081  AF263082 | EF672422  EF672423 |  |  | [33]  [33] |
| *Cobitis fahireae* |  | 1TK  2TK  373TR | R. Bakir, Pergamon (=Bergama), Izmir, TR  R. Bakir, Pergamon, Izmir,TR  R. Bakir, Pergamon, Izmir,TR | EF672387  EF672388  DQ217395 | EF672424  EF672425  KP161147 |  |  | [33]  [33]  [19] |
| *Cobitis hankugensis* |  | A2020  A2021 | Geum Cheon, Geum, Nae Hoa, KR  Geum Cheon, Geum, Nae Hoa, KR | KP161086  KP161087 | KP161148  KP161149 |  |  |  |
| *Cobitis hellenica* |  | 96G  156G | R. Thiamis, Thiamis, Parapotamus, GR  R. Louros, Louros, Agio Georgios, GR | AF263087  AY191583 | EF672426  EF672427 |  |  | [19]  [19] |
| *Cobitis illyrica* |  | BOS16C  BOS129  BOS140A  BOS140B | Lake Krenica, Krenica, Matica, BA  Mostarsko Blato, Mostarsko Blato, Listica, BA  Mostarsko Blato, Mostarsko Blato, Listica, BA  Mostarsko Blato, Mostarsko Blato, Listica, BA | EF672404  EF672406  EF672407 | EF672434  EF67243  EF672435  KP161150 |  |  | [33] (as *Cobitis* sp. A)  [33] (as *Cobitis* sp. A)  [33] (as *Cobitis* sp. A)  [33] (as *Cobitis* sp. A) |
| *Cobitis jadovaensis* |  | CBJA1-3 | R. Jadova, Jadova, HR | EF605307 |  |  |  | [55] |
| *Cobitis laoensis* |  | 383  384 | Unknown river, central Vietnam, VN  Unknown river, central Vietnam, VN | KP161088  KP161089 |  |  |  |  |
| *Cobitis lebedevi* |  | 3585DPT  3586APT  CMK19607 | R. Tunguska, lower Amur, Khabarovsk Dist., RU  R. Arsenyevka, Ussury, Ozernoye, Primorye Dist., RU  R. Dund Bayen, Khentii, MN | JN858871  JN858872  KP161090 | JN858826  JN858827 |  |  | [25] (as *C. choii*)  [25] (as *C. choii*) |
| *Cobitis* cf. *lebedevi* |  |  | Haerbin, Heilongjiang, CN | DQ105242 |  |  |  | [27] (as *Cobitis granoei*) |
| *Cobitis levantina* |  | 380  382 | Channel, Orontes, Hatay, TR  Channel, Orontes, TR | DQ217386  DQ217387 |  |  |  | [19]  [19] |
| *Cobitis linea* |  | 4618  4619  4620 | Creek, Kor, Doroodzan, IR  Creek, Kor, Doroodzan, IR  Creek, Kor, Doroodzan, IR | DQ217388  DQ217389  DQ217390 | KP161151  KP161152  KP161153 |  |  | [19]  [19]  [19] |
| *Cobitis lutheri* |  | 3621PT  3649PT | R. Karasik, Tumannaya, Primorye Dist., RU  R. Gur, lower Amur, Voskresensk,Khabarovsk Dist., RU | JN858891  JN858893 | JN858843  JN858844 |  |  | [25]  [25] |
| *Cobitis macrostigma* |  | H1  H2 | Hukou, Jiangxi, CN  Yueyang, Hunan, CN | DQ105229  DQ105230 |  |  |  | [27]  [27] |
| *Cobitis maroccana* |  | 8CMKHE  10CMKHE | R. Kherrouba, Sebou, Kherrouba, MA  R. Kherrouba, Sebou, Kherrouba, MA | KP161091  KP161092 | KP161154  KP161155 |  |  |  |
| *Cobitis gladkovi** | *C melanoleuca** | Mosk132  Don369  1187MO  1188MO  A1067  A1068  CMK19607 | R. Moskwa, Volga, Moscow, RU  R. Don, Don, Rostov, RU  R.Sinukha, Kuban, Krasnodarski, RU  R.Sinukha, Kuban, Krasnodarski, RU  R. Don, Don, Rostov, RU  R. Don, Don, Rostov, RU | KP161093  KP161094  AF263068 | EF672428  KP161156  EF508615  EF056343 |  |  | [21] (*as C. cf. sibirica*),  [17] (as *C. melanoleuca*)  [17] (as *C. melanoleuca*); [26] (as *C. melanoleuca*) |
| *Cobitis sibirica** | *C melanoleuca** | CMK19644  CMK195781  CMK195782 | R. Khurkh, Khentii, MN  R. Bulgan Gol, Khovd, MN  R. Bulgan Gol, Khovd, MN | KP161095  KP161096  KP161097 |  |  |  |  |
| *Cobitis meridionalis* |  | 1273g(=117SAB)  1276g(=120SAB) | Lake Prespa, Prespa, Psarades, GR  Lake Prespa, Prespa, Psarades, GR | AF263083  AF263084 | KP161157  EF672429 |  |  | [21]  [21] |
| *Cobitis narentana* |  | BOS620C  BOS620E  BOS620H  BOS217A | R. Tebrinisca, Tebrinisca, Ravno, BA  R. Tebrinisca, Tebrinisca, Ravno, BA  R. Tebrinisca, Tebrinisca, Ravno, BA  Hutovo Blato, Neretva, Gnijilista, BA | EF672391  EF672392 | KP161158  EF672430  KP161159  KP161160 |  |  | [33]  [33] |
| *Cobitis ohridana* |  | CO20  CO40  A590  A591  A595  BOS967  G200 | Lake Ohrid, Ohrid, Bugez, AL  R. Moraca, Skadar, Podgorica, ME  R. Erzen, Erzen, Mullet, AL  R. Erzen, Erzen, Mullet, AL  R. Drinos, Aoos, Epirus, GR  Lake Ohrid, Ohrid, Buqez, AL  R. Aoos, Aoos, Konitsa, GR | EF597227  EF597229  EF672397  EF672398  EF672399  EF672394  AY191563 | EF672431  KP161161  EF672432  KT717937  EF672433 |  |  | [45]  [45]  [33]  [33]  [33]  [33]  [33] |
| *Cobitis paludica* |  | 17LAGRUI  584CPTala | Ruidera Lagoons, Guadiana, ES  Fuente del Roble Lagoon, Tajo, Talayuela, ES | KP161098  AF263074 | KP161162  KP161163 |  |  | [21] |
| *Cobitis phrygica* |  | 28TR | Gemic spring, Lake Aci Golu, Denizli,TR | DQ217383 |  |  |  | [19] (as *C. turcica*) |
| *Cobitis pontica* |  | KJ2004 E22 | R. Veleka, Veleka, Burgas, BG | AY706184 |  |  |  | [56] (as *Cobitis* sp.) |
| *Cobitis punctilineata* |  | 1507G  1509G  1511G | Channel of R. Angitis, Strymon, Fotolivos, GR  Channel of R. Angitis, Strymon, Fotolivos, GR  Channel of R. Angitis, Strymon, Fotolivos, GR | KP161099  KP161100  KP161101 | KP161164  KP161165  KP161166 |  |  |  |
| *Cobitis rara* |  | A2019  374  375 | Ornamental fish trade, CN  Ornamental fish trade, CN  Ornamental fish trade, CN | EF508507  KP161102  KP161103 | EF508619 |  |  | [17] |
| *Cobitis satunini* |  | A2050  A2051  A2052  A2053  JB2005 | Unknown river, Calidabad, AZ  Unknown river, Calidabad, AZ  Unknown river, Calidabad, AZ  Unknown river, Calidabad, AZ  R. Rioni, Rioni, Imereti, GE | KP161104  KP161105  KP161106  KP161107  DQ217396 | KP161167  KP161168  KP161169 |  |  | [19] (as *Cobitis* sp. Rioni) |
| *Cobitis shikokuensis* |  | Chr284 | R. Hiji, Ehime, Shikoku, JP | AP009306 | AB531329-30 |  |  | [54] |
| *Cobitis sinensis* |  | 1  Cob5  2  3 | Guangxi, CN  Unknown source  Unknown source  Unknown source | AY625699  AY281266  AY526868  NC_007229 |  |  |  | Wang & Tzeng (unpub)  Chen & Wang (unpub)  Chen & Wang (unpub) |
| *Cobitis* cf *sinensis* |  | IHCAS8  IHCAS11  IHCAS13 | Jiangxi, Wuyuan, CN  Jiangxi, Shangrao, CN  Chenxi, Hunan, CN | DQ105234  DQ105233  DQ105235 |  |  |  | [27]  [27]  [27] |
|  | *Cobitis* sp. aff. *lutheri* | A2023  A2024  A2026 | R. Jin Am Gang, Yeongsan, Oa U, KR  R. Jin Am Gang, Yeongsan, Oa U, KR  R. Jin Am Gang, Yeongsan, Oa U, KR | KP161108  KP161109 | KP161170  KP161171  KP161172 |  |  |  |
|  | *Cobitis* sp. B | A585  A592  345 | Lake Ohrid, Ohrid, Buqez, AL  Lake Skadar, Skadar, Shiroke, AL  R. Moraca, Skadar, Podgorica, ME | EF672408  EF672409  EF672410 | EF672436  EF672437 |  |  | [33]  [33]  [33] |
|  | *Cobitis* sp. CHI | China1  China3 | Ornamental fish trade, CN  Ornamental fish trade, CN | KP161110  KP161111 |  |  |  |  |
|  | *Cobitis* sp. Vietnam | Vietnam1  Vietnam2 | R. Ky Cung, Pearl, Lang Son, VN  R. Ky Cung, Pearl, Lang Son, VN | KP161112  KP161113 |  |  |  |  |
|  | *Cobitis* sp. Yangtze | Yangtze1  Yangtze2 | R. Yangtze, Yangtze, Wuhan, CN  R. Yangtze, Yangtze, Wuhan, CN | KP161114  KP161115 |  |  |  |  |
| *Cobitis splendens* |  | 1TK  2TK | Duzce, TR  Duzce, TR | KP161116  KP161117 | KP161173  KP161174 |  |  |  |
| *Cobitis stephanidisi* |  | 743G  744G  745G  746G | Velestino spring, Velestino, Thessaly, GR  Velestino spring, Velestino, Thessaly, GR  Velestino spring, Velestino, Thessaly, GR  Velestino spring, Velestino, Thessaly, GR | KP161118  KP161119 | KP161175  KP161176  KP161177  KP161178 |  |  |  |
| *Cobitis striata* |  | 4n  2n | Lake Biwa, Hamabum, Shiga, Honshu, JP  Irrigation creek, Takashima, Makino, Shiga, Honshu, JP | AB054125  AP010782 | AB373204  AB531315-16 |  |  | [57] [58]  [54] |
| *Cobitis strumicae* |  | 1428G  1659G | R. Strymon, Strymon, Strimoniko, GR  R: Macropotamus, Filiouris, Gratini, GR | AY191578  AY191574 | KP161179  KP161180 |  |  | [21]  [21] |
| *Cobitis taenia* |  | 1TAE  A1860 | R. Hunte, Weser, Niedersachsen, DE  R. Hunte, Weser, Niedersachsen, DE | AF263077  EF508508 | EF672438  EF056334 |  |  | [21]  [17] |
|  | *Cobitis* indet. | IHCAS17 | China | DQ105244 |  |  |  | [27] (as *Cobitis* cf. *taenia*) |
| *Cobitis takatsuensis* |  | Chr284 | R. Misumi, Honshu, JP  R. Tsutsuga, Ota, Honshu, JP  R. Hiji, Ehime Prefecture, Shikoku, JP | AB039337  AB039338  NC015306 | EU409616 |  |  | [59]  [56]  [54] |
| *Cobitis tanaitica* |  | TAN1  TAN3 | Lake Sinoe, Sinoe, Sinoe, RO  Lake Sinoe, Sinoe, Sinoe, RO | DQ217397  DQ217398 | KP161181  KP161182 |  |  | [19]  [19] |
| *Cobitis taurica* |  | CtauT6  CtauT7  CtauT8 | R. Savranka, Southern Bug, Odessa, UA  R. Chernaya, Chernaya, Crimean Peninsula, UA  R. Chernaya, Chernaya, Crimean Peninsula, UA | AY706162  AY706165 AY706166 |  |  |  | [56]  [56]  [56] |
| *Cobitis tetralineata* |  | A1922  A1923  A1924 | R. Seom Jin Gang, Seomjin, Gye San, KR  R. Seom Jin Gang, Seomjin, Gye San, KR  R. Seom Jin Gang, Seomjin, Gye San, KR | KP161120  KP161121 | KP161183  KP161184  KP161185 |  |  |  |
| *Cobitis trichonica* |  | 1178G  1179G | Lake Trichonis, Trichonis, Panetolio, GR  Lake Trichonis, Trichonis, Panetolio, GR | AF263085  AF263086 | EF672440  EF672473 |  |  | [21]  [21] |
| *Cobitis turcica* |  | 223TR  364TR  226TR  A2235  A2236 | Lake Beysehir, Beysehir, Konya,TR  Lake Beysehir, Beysehir, Konya,TR  creek at Duger, Burdur, Burdur, TR  R. Kirk Göz, Kirk Göz, Antalya, TR  Lake Beysehir, Beysehir, Konya, TR | DQ217379  DQ217377  DQ217381 | KP161186  KP161187  EF508620  EF508621 |  |  | [19]  [19]  [19]  [17]  [17] |
| *Cobitis vardarensis* |  | 323G  370G | R. Agiaki, Vardar, Kastanies, GR  R. Agiaki, Vardar, Kastanies, GR | AF263079  AF263080 | EF672442  EF672443 |  |  | [21]  [21] |
| *Cobitis vettonica* |  | 5ARR  158CP | R. Arrago, Tajo, Cadalso, ES  Malena Stream, Duero, Cáceres, ES | KP161122  KP161123 | KP161188  KP161189 |  |  |  |
| *Cobitis zanandreai* |  | 17G  19G  36G  37G  39G | Lake Fondi, Fondi, San Rafaele, IT  Lake Fondi, Fondi, San Rafaele, IT  R. Vedere, Fondi, Fondi, IT  R. Vedere, Fondi, Fondi, IT  R. Vedere, Fondi, Fondi, IT | EF672411  KP161124  EF672412  KP161125 | KP161190  KP161191  KP161192  KP161193  KP161194 |  |  | [21]  [21] |
| *Iksookimia hugowolfeldi* |  | Cob_6 | Daedong Dam, Yongsan, Jeonnam, KR | EU670758 |  |  |  | Bang & Lee (unpub) |
| *Iksookimia koreensis* |  | A1868  A1869 | R. Han Tan, Han, Cheon Gon, KR  R. Han Tan, Han, Cheon Gon, KR | EF508511  EF508512 | EF508623  EF508624 |  |  | [17]  [17] |
| *Iksookimia longicorpus* |  | A1918  A1919 | R. Seom Jin, Seom Jin, Gye San, KR  R. Seom Jin, Seom Jin, Gye San, KR | EF508513  EF508626 | EF508625  EF508626 |  |  | [17]  [17] |
| *Iksookimia pacifica* |  | A2000  A2001  A2002 | Cheon Jin, Cheon Jin, Cheon Jin, KR  Cheon Jin, Cheon Jin, Cheon Jin, KR  Cheon Jin, Cheon Jin, Cheon Jin, KR | KP161126  KP161127  KP161128 | KP161195  KP161196  KP161197 |  |  |  |
| *Iksookimia pumila* |  | A1927  A1928 | R. Baeg, Baeg, Cheong Rim, KR  R. Baeg, Baeg, Cheong Rim, KR | EU67076  EF508515 | EF508627  EF508628 |  |  | [17]  [17] |
| *Iksookimia yongdokensis* |  | A1896  A1897 | R. Dae Seo, Osip, Yong Pyeong, KR  R. Dae Seo, Osip, Yong Pyeong, KR | EF508516  EF508517 | EF508629  EF508630 |  |  | [17]  [17] |
| *Kichulchoia brevifasciata* |  | A1936  A1937 | R. Ko Eub, Ko Eub, Ya Mag, KR  R. Ko Eub, Ko Eub, Ya Mag, KR | EF508518  EF508519 | EF523406  EF523407 |  |  | [17]  [17] |
| *Kichulchoia multifasciata* |  | Cob-16  A1908  A1907 | Daepyeong-myeon, KR  R. Geum, Geum, Hyeong, KR  R. Geum, Geum, Hyeong, KR | EU670768  EF508573  EF508574 | EU670844  EF508663 |  |  | [60]  [17]  [17] |
| *Koreocobitis naktongensis* |  | A1970  A1971 | R. Geu, Naktong, Nae Hoa, KR  R. Geu, Naktong, Nae Hoa, KR | EU670764  EF508520 | EF508631  EF508632 |  |  | [17]  [17] |
| *Koreocobitis rotundicaudata* |  | A1886  A1965 | R. Jeon, Han, Gyo Hang, KR  R. Jeon, Han, Gyo Hang, KR | EF508521  EF508522 | EF508632  EF508633 |  |  | [17]  [17] |
| *Kottelatlimia pristes* |  | A1626 | Forest stream, Sarawak, Sarawak, MY | EF508546 | EF508638 |  |  | [17] |
| *Microcobitis misgurnoides* |  | 124  125  127  A300_CM1  A301_CM2  A357_CM3  A358_CM4 | R. Thua Luu, Thua Luu, Thua Thin Hue, VN  R. Thua Luu, Thua Luu, Thua Thin Hue, VN  R. Thua Luu, Thua Luu, Thua Thin Hue, VN  R. Thua Luu, Thua Luu, Thua Thin Hue, VN  R. Thua Luu, Thua Luu, Thua Thin Hue, VN  R. Thua Luu, Thua Luu, Thua Thin Hue, VN  R. Thua Luu, Thua Luu, Thua Thin Hue, VN | KP161129  KP161130  KP161131  EF508501  EF508502  EF508503  EF508504 | EF508616 |  |  | [17]  [17]  [17]  [17] |
| *Microcobitis sp.* |  | CMK 201011  CMK 201012 | Nong Bocho, Mekong, Khammouan, LA  Nong Bocho, Mekong, Khammouan, LA | KP161132  KP161133 |  |  |  |  |
| *Misgurnus anguillicaudatus* |  | A406  A408  A415  A1847 | Stream in Imaizumi, Natori, Miyagi, JP  Stream in Imaizumi, Natori, Miyagi, JP  unnamed stream, Yangtze, Wuhan, CN  No details known, Taiwan, TW | EF508556  EF508558 | EF508651  EF056344  EF508652 |  |  | [17]  [17]  [17]  [17] |
| *Paramisgurnus dabryianus,*  *Misgurnus mizolepis* |  | A409  A412  Cob_15 | Unnamed stream, Yangtze, Wuhan, CN  Ornamental fish trade  Biin-myeon, KR | EF508591  EF508589  EU670767 | EF508675  EF508676  EU670843 |  |  | [17]  [17]  [60] (as *Misgurnus mizolepis*) |
| *Misgurnus fossilis* |  | A081  A683  A464 | Unnamed channel, Vistula, Bus’k, UA  Small stream, unknown drainage, Zhytomyr, UA  Lake Jeglowek, Niemen, Warmia-Masuria, PL | EF508560  EF508563 | EF508655  EF508654 |  |  | [17]  [17]  [17] |
| *Misgurnus mohoity* |  | 3603APT  3605PT | R. Ilya, upper Amur, Chita, RU  Unnamed spring, lower Amur, Khabarovsk, RU | JN858850  JN858853 | JN858807  JN858810 |  |  | [25]  [25] |
| *Misgurnus nikolskyi* |  | 3580APT  3633PT | R. Kupriyanikha, lower Amur, Khabarovsk, RU  Grishka’ channel, Tumannaya, Primorye, RU | JN858854  JN858864 | JN858811  JN858819 |  |  | [25]  [25] |
| *Niwaella delicata* |  | A2177  A2178 | R. Takahara, Jinzu, Gifu, JP  R. Obora, Jinzu, Gifu, JP | EF508571  EF508572 | EF508661  EF508662 |  |  | [17]  [17] |
| *Niwaella cf. laterimaculata* |  | IHCAS009 | Wuyuan, Jiangxi, CN | DQ105236 |  |  |  | [27] |
| *Pangio pangia* |  | A1779 | R. Tista, Tista, West Bengal, IN | EF508583 | EF508670 |  |  | [17] |
| *Sabanejewia aurata* |  | A1843  245 | R. Arax, Arax, Kar, TR  R. Rioni, Rioni, GE | EF508593  AF499189 | EF508677 |  |  | [17]  [22] |
| *Sabanejewia balcanica* |  | 41RU  48RU  G401  G453  436G  459CRR  1732G  1775G | R. Timis, Danube, Çag, RO  R. Timis, Danube, Çag, RO  R. Agiaki, Doirani, West Macedonia, GR  Lake Doirani, Doirani, West Macedonia, GR  R. Mures, Danube, Gornesti, RO  R. Mures, Danube, Pecica, RO  R. Erythropotamus, Evros, Mikro Derio, GR  R. Erythropotamus, Evros, Mikro Derio, GR | KP161134  KP161135  AY059356  AY059354  AY059360  AY059357  AY059362  AY059363 | KP161198  KP161199  KP161200  KP161201  KP161202  KP161203  KP161204 |  |  | [22] (as *S. doiranica*)  [22] (as *S. doiranica*)  [22] (as *S. radnensis*)  [22] (as *S. radnensis*)  [22] (as *S. thrakica*)  [22] (as *S. thrakica*) |
| *Sabanejewia baltica* |  | 115MNCN  116MNCN | R. Bug, Vistula, Biala Podlaska, PL  R. Bug, Vistula, Biala Podlaska, PL | AY059340  AY059341 | KT717938 |  |  | [22]  [22] |
| *Sabanejewia bulgarica* |  | 166  218CRB | R. Danube, Danube, Polizesi, RO  R. Cerna, Danube, Orsova, RO | AF499188  AY059353 | KT717939  KT717940 |  |  | [22]  [22] |
| *Sabanejewia caucasica* |  | 257MO  258MO  260MO | R. Kuma, Kuma, Stravropolskij Kraj, RU  R. Kuma, Kuma, Stravropolskij Kraj, RU  R. Kuma, Kuma, Stravropolskij Kraj, RU | AY059338  AY059339 | KT717941  KT717942 |  |  | [22]  [22] |
| *Sabanejewia kubanica* |  | 1Kuban27.1.1  2Kuban27.1.2  3Kuban27.1.3 | R. Kuban, Kuban, Krasnodar, RS  R. Kuban, Kuban, Krasnodar, RS  R. Kuban, Kuban, Krasnodar, RS | AF499182  AF499184 | KT717943  KT717944  KT717945 |  |  | [22] (as S. aurata kubanica)  [22] (as S. aurata kubanica)  [22] (as S. aurata kubanica) |
| *Sabanejewia larvata* |  | A1859  41TA  198TA  244TA | Unnamed stream, Po, Venice, IT  R. Melleta, Po, Carmagnola, IT  R. Melleta, Po, Bossano, IT  Fosseta Armi, Po, Crevalcore, IT | EF508594  AY059335 | KT717946  KT717947  KT717948 | 44°51'7.60"N  44°41'5.41"N | 7°41'2.56"E  7°40'22.19"E | [17]  [22] |
| *Sabanejewia romanica* |  | A1857  74G  213CRO | R. Oltul, Danube. Olt, RO  R. Dîmbovitza, Danube, Dragomiresti, RO  R. Valsan, Danube, Valsanesti, RO | EF508595  AF499196 | EF056338  KT717949 |  |  | [17]  [21] |
| *Sabanejewia vallachica* |  | 24RU  32RU  81CRU  100CRU | R. Ialomitza, Danube, Tiganesti, RO  R. Ialomitza, Danube, Tiganesti, RO  R. Dîmbovitza, Danube, Dragomiresti, RO  R. Dîmbovitza, Danube, Dragomiresti, RO | AY059368  AY059370 | KT717950  KT717951  KT717952 |  |  | [22]  [22] |

* *Cobitis melanoleuca* was subdivided in 3 subspecies (*C. melanoleuca melanoleuca, C. m. gladkovi, and C. m. granoei*) according to Vasil’ev & Vasil’eva 2008. Kottelat 2012 treated all these *C. melanoleuca* subspecies as distinct species: *C. melanoleuca*, *C. gladkovi* and *C. sibirica* (considering *C. m. granoei* a synonym of *C. sibirica*) arguing that they satisfy the criteria of species under the Evolutionary Species Concept but without any convincing evidence to be considered good species. In our study, we have maintained *C. melanoleuca* as a good species in all analyses (see results) based on the study of Perdices et al. 2015 to avoid further taxonomic confusion until revision.

54. Saitoh K, Chen WJ, Mayden RL. Extensive hybridization and tetrapolyploidy in spined loach fish. Mol Phylogenet Evol. 2010;56: 1001-1010.

55. Buj I, Podnar M, Mrakovčič M, Choleva L, Šlechtová V, Tvrtković N, et al. Genetic diversity and phylogenetic relationships of spined loaches (genus *Cobitis*) in Croatia based on mtDNA and allozyme analyses. Folia Zool. 2008*;*57 (1-2): 71-82.

56. Janko K, Culling MA, Rab P, Kotlik P. Ice age cloning - comparison of the Quaternary evolutionary histories of sexual and clonal forms of spiny loaches (*Cobitis*; Teleostei) using the analysis of mitochondrial DNA variation. Mol Ecol*.* 2005;14: 2991-3004.

57. Saitoh K, Miya M, Inoue JG, Ishiguro NB, Nishida M. Mitochondrial genomics of ostariophysan fishes: perspectives on phylogeny and biogeography. J Mol Evol. 2003*;* 56: 464-472.

58. Saitoh K, Chen WJ. Reducing cloning artifacts for recovery of allelic sequences by T7 endonuclease I cleavage and single re-extension of PCR products-a benchmark. Gene*.* 2008;423: 92-95.

59. Kitagawa T, Watanabe M, Kobayashi T, Yoshioka M, Kashiwagi M, Okazaki T. Two genetically divergent groups in the Japanese spined loach, *Cobitis takatsuensis*, and their phylogenetic relationships among Japanese *Cobitis* inferred from mitochondrial DNA analyses. Zool Science. 2001;18: 249-259.

60. Lee IR. Studies on the conservation biology of an endangered freshwater fish, *Iksookimia choii*. Ph.D. Thesis, Soonchunhyang University. Asan, South Korea. 2009.
